# Cumulative Merging Percolation and the Epidemic Transition of the Susceptible-Infected-Susceptible Model in Networks

**DOI:** 10.1103/PhysRevX.10.011070

**Published:** 2020-03-24

**Authors:** Claudio Castellano, Romualdo Pastor-Satorras

**Affiliations:** ^1^ Istituto dei Sistemi Complessi (ISC-CNR), Via dei Taurini 19, I-00185 Roma, Italy; ^2^ Departament de Física, Universitat Politècnica de Catalunya, Campus Nord B4, 08034 Barcelona, Spain

## Abstract

We consider cumulative merging percolation (CMP), a long-range percolation process describing the iterative merging of clusters in networks, depending on their mass and mutual distance. For a specific class of CMP processes, which represents a generalization of degree-ordered percolation, we derive a scaling solution on uncorrelated complex networks, unveiling the existence of diverse mechanisms leading to the formation of a percolating cluster. The scaling solution accurately reproduces universal properties of the transition. This finding is used to infer the critical properties of the susceptible-infected-susceptible model for epidemics in infinite and finite power-law distributed networks. Here, discrepancies between analytical approaches and numerical results regarding the finite-size scaling of the epidemic threshold are a crucial open issue in the literature. We find that the scaling exponent assumes a nontrivial value during a long preasymptotic regime. We calculate this value, finding good agreement with numerical evidence. We also show that the crossover to the true asymptotic regime occurs for sizes much beyond currently feasible simulations. Our findings allow us to rationalize and reconcile all previously published results (both analytical and numerical), thus ending a long-standing debate.

## Summary

Complex networks represent the interaction pattern for many real-world phenomena such as epidemic spreading. The simplest and most fundamental model for the diffusion of infectious diseases without acquired immunity predicts a vanishing epidemic threshold in the limit of large systems. In other words, no matter how small the infectiousness of the disease, there is always a finite fraction of the overall population which is infected for long times. So far, the detailed mechanism underlying this phenomenology has remained unclear. Here, we provide a complete and quantitative understanding of the model’s behavior.

While the role of hubs (individuals with high connectivity) was already believed to be important, we fully clarify its nature, pointing out the highly nontrivial interplay among hubs. Each hub acts as an infection hotbed and the global epidemic emerges from hubs reinfecting each other. Surprisingly, this mechanism is at work even when the hubs are not in direct mutual contact but transmit the infection through chains of low-connectivity individuals. The survival of the epidemic is then a manifestation of a novel, long-range, percolation process among distant hubs, whose properties explain the behavior of the epidemic model.

These findings rationalize and reconcile previously published results and point out long-range indirect interactions as potentially crucial ingredients for other collective phenomena in networked systems.

## I. INTRODUCTION

Percolation and epidemic spreading are among the most interesting processes unfolding on complex network substrates, and their investigation has attracted a huge interest in the past 20 years [1–5]. One of the most successful achievements of this endeavor is the realization that the properties of one of the fundamental models for epidemics without a steady state, the susceptible-infected-recovered (SIR) dynamics [6], can be mapped onto bond percolation [7–9]. This connection has permitted the application to the SIR model of the powerful tools devised for percolation, leading to a full understanding of this epidemic process [8,10–13]. For the other fundamental class of epidemic dynamics, allowing for a steady, endemic state, whose simplest representative is the susceptible-infected-susceptible (SIS) model [6], no direct mapping to a percolative framework is available, and theoretical progress has been slower. In the SIS model, susceptible individuals acquire the disease at rate β through any edge connected to an infected individual, while infected individuals spontaneously heal with rate μ. The epidemic threshold $\lambda_{c}$ defines the value of the ratio λ=β/μ separating a healthy (absorbing) phase from an endemic one with everlasting activity. Initial work showed that degree heterogeneity leads to disruptive effects on scale-free networks [14], namely, a vanishing threshold in networks with power-law degree distribution $P(k)∼k^{-\gamma }$ and γ≤3 [15,16]. Later efforts have shifted toward less heterogeneous networks, those with γ>3 [3].

The quenched mean-field (QMF) theory [17–19] predicts a vanishing threshold $\lambda_{c}\to 0$ in the infinite network-size limit for any value of γ [20], due to the existence of hubs able to sustain the epidemic for long times only by interacting with their direct neighbors [21]. It was later pointed out that, at the QMF level, the localization of activity around these hubs implies the existence, for small values of λ, of long-lived, but not stationary, states [22,23]. Important progress in this debate is provided in Ref. [24], where it is shown that a genuine non-mean-field effect, mutual reinfection among distant hubs, is the key mechanism triggering the appearance of an endemic stationary state for any λ in networks with γ>5/2. Numerical evidence corroborates this picture, showing that the position of the effective threshold tends to zero with network size for any γ. However, the decay observed is slower than the one predicted by the QMF theory [24–26] and, moreover, in contradiction with recent mathematical results derived by Huang and Durrett [27] and Mountford, Valesin, and Yao [28]. An additional puzzling question in this area is the striking disagreement between the exact mathematical prediction for the singular behavior of the prevalence ($\rho ∼\lambda^{2\gamma -3}$, apart from logarithmic corrections) [28] and numerical simulations exhibiting a much faster growth. This lack of a precise agreement between analytics and numerics represents one standing issue in our understanding of epidemic processes on complex topologies.

A precise mathematical formulation of the mutual reinfection process was recently proposed by Ménard and Singh [29]. They introduce the cumulative merging percolation (CMP) process, a long-range site percolation process [30] aimed at describing the geometry of the sets where SIS epidemics survive for a long time on a network. The presence of a CMP giant component corresponds to the existence of an endemic SIS stationary state, so that the calculation of the CMP threshold allows one to locate also the position of the SIS epidemic transition [31].

In this paper, we contribute to the current state-of-the-art in this area in two ways. First, we consider a generalized version of the CMP process proposed in Ref. [29], and we present a scaling theory for its nontrivial behavior. This theory—which provides a clear understanding of competing physical mechanisms, critical properties, crossover scales, and finite-size effects—is general and can be related with other processes. Second, we apply the results of the first part to SIS dynamics, obtaining in this way for the first time a full understanding of the critical properties of the model. In particular, our theory predicts that the asymptotic behavior in the limit of very large networks (derived exactly in Refs. [27,28] and reassuringly recovered by our approach) can be observed only for huge system sizes, out of reach for present computer resources. We show instead that, for network sizes that can be currently simulated, a preasymptotic regime holds, whose nontrivial properties are determined, providing a prediction for the finite-size scaling of the SIS epidemic threshold in agreement with (previously unexplained) numerical results. Our work reconciles in a comprehensive way the different theories proposed to interpret the behavior of the SIS model, placing them in the proper context regarding the network size considered, and thus ends a long debate between the physics and mathematics communities.

The paper is organized as follows: In Sec. II, we define the cumulative merging percolation process which is the subject of our study. Section III presents a scaling solution of this model, whose behavior in finite networks is discussed in Sec. IV. A numerical check of the scaling solution is provided in Sec. V. In Sec. VI, we apply the results obtained to the SIS epidemic model, backing up our conclusions by comparison with existing numerical simulations. Finally, in Sec. VII, we summarize our main results and discuss the interesting perspectives they open. Several Appendixes provide some detailed analytical calculations and additional information.

## II. CUMULATIVE MERGING PERCOLATION PROCESS

**FIG. 1.**
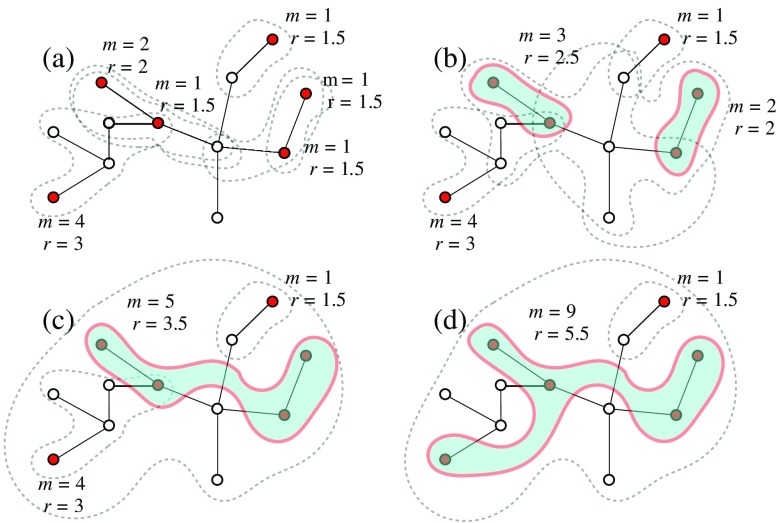
Schematic illustration of the CMP process for r(m)=1+m/2. Filled nodes are active; empty nodes are inactive. Areas bordered by dashed lines are interaction domains of active nodes or clusters. Clusters are indicated by solid lines surrounding a filled area. Notice in (c) and (d) the isolated active node on the upper right corner, which is within the interaction range of the large cluster but cannot be merged as it has r<2.

We consider a generalization of the cumulative merging process proposed in Ref. [29], defined along the following lines. In a given network, composed by N nodes, each node i is *active* with probability $p_{i}$. Inactive nodes do not play any role apart from determining the topological distances between pairs of active nodes (see below). Each active node i defines a cluster of size 1, associated with an initial mass $m_{i}^{\left(0\right)}$. Starting with these initial clusters, an iterative process takes place whose elementary step is the merging of a pair of clusters into a single one. Two clusters, α and β, are merged in a single cluster if there are at least a node $i_{\alpha }$ in α and a node $j_{\beta }$ in β, such that

$$d_{i_{\alpha },j_{\beta }}\leq \min\{r(m_{\alpha }),r(m_{\beta })\},$$ (1)

where $d_{i,j}$ is the topological distance between nodes i and j and r(m)≥1 is an interaction range associated to a cluster of mass m. The mass of the merged cluster is the sum of the masses of the original clusters: $m_{\alpha +\beta }=m_{\alpha }+m_{\beta }$. The iteration of this procedure converges to a limiting partition of the network that does not depend on the order in which the merging is performed [32]. Notice that if $p_{i}=p$ and r(m)=1, CMP coincides with random site percolation [1]. It is important to remark that Eq. (1) implies that two clusters merge only if each one of them is within the interaction range of the other: An asymmetric situation, with a massive cluster interacting with a far and small cluster, does not lead to merging. In Fig. 1, we present a graphical illustration of the mechanism of the CMP process. We stress again that a cluster is defined as a set of (only) active nodes resulting from the iteration of merging events. Nodes in the same cluster must belong to the same connected component of the underlying network, but they do *not* need to form a connected component by themselves, as is clear from Figs. 1(c) and 1(d).

The connection between CMP and the mutual reinfection of distant hubs in the SIS epidemics is operated by taking as active nodes the hubs able to independently sustain the epidemic [29]; see Appendix A for a detailed description.

## III. SCALING THEORY FOR CUMULATIVE MERGING PERCOLATION

Let us focus now on a specific yet broad class of CMP processes, where nodes are active if their degree is larger than a threshold value $k_{a}$: $p_{i}\equiv p(k_{i})=\Theta (k_{i}-k_{a})$. In an uncorrelated network with degree distribution $P(k)=(\gamma -1)k_{\min}^{\gamma -1}k^{-\gamma }$ in the continuous approximation, where $k_{\min}$ is the minimum degree, the fraction of active nodes is

$$\frac{N_{a}}{N}=\int_{k_{a}}^{\infty }dkP(k)={\left(\frac{k_{a}}{k_{\min}}\right)}^{1-\gamma }.$$ (2)

We are interested in understanding the possible existence of a CMP giant component as a function of $k_{a}$, in particular, in the limit $k_{a}\to \infty$, when only a small fraction of nodes is active.

### A. The case r(m)=1: Degree-ordered percolation

Let us consider first the case r(m)=1, i.e., only nearest neighbors can form clusters. In this case, the CMP process defined above coincides with the degree-ordered percolation (DOP) process proposed in Ref. [23] (coinciding with the limit α→-∞ in Ref. [33]). For a node of degree k, the probability that a given neighbor is active is

$$P_{a}(k)=\int_{k_{a}}^{\infty }dk^{\prime }P(k^{\prime }|k),$$ (3)

where $P(k^{\prime }|k)$ is the conditional probability that a neighbor of a node k has degree $k^{\prime }$ [34]. For uncorrelated networks, $P(k^{\prime }|k)=(k^{\prime }P(k^{\prime })/\langle k\rangle )$ [34]; thus, we have $P_{a}={\left(k_{a}/k_{\min}\right)}^{2-\gamma }$, independent of k. The mean number of active neighbors of a node of degree k is $kP_{a}$; therefore, the inverse of $P_{a}$,

$$k_{c}={\left(\frac{k_{\min}}{k_{a}}\right)}^{2-\gamma },$$ (4)

defines a degree scale separating nodes likely to have many active neighbors $k/k_{c}\gg 1$ from those likely to be *isolated*, i.e., not in direct contact with any active node. The average number of active neighbors for each active node is

$$\frac{N}{N_{a}}\int_{k_{a}}^{\infty }dkP(k)kP_{a}=\frac{\gamma -1}{\gamma -2}k_{a}P_{a}∼k_{a}^{3-\gamma }.$$ (5)

For γ<3, this quantity diverges as $k_{a}$ grows: Each active node has a very large number of active neighbors, so that all of them belong to a connected giant component for any $k_{a}$ [23,33], and the relative size S of the giant component is simply given by the fraction of active nodes

$$S_{\mathrm{DOP}}=\frac{N_{a}}{N}={\left(\frac{k_{a}}{k_{\min}}\right)}^{1-\gamma }.$$ (6)

For γ>3, instead, the average number of active neighbors of an active node decreases with $k_{a}$ and tends to zero in the limit $k_{a}\to \infty$. This result indicates that a degree-ordered percolation giant component (DOPGC) can exist only up to a finite threshold value, in agreement with Refs. [23,33]. It is useful to discuss the behavior of the order parameter $S_{\mathrm{DOP}}$ as a function of $k_{a}$ in this case. For $k_{a}=k_{\min}$, $k_{c}=1$. Hence, even for γ>3, there is an interval of $k_{a}$ values such that $k_{a}/k_{c}>1$. This regime occurs up to a value $k_{a}=k_{0}^{*}$ determined by the condition $k_{c}(k_{0}^{*})=k_{0}^{*}$, yielding

$$k_{0}^{*}=k_{\min}^{\left(\gamma -2)/(\gamma -3\right)}.$$ (7)

Notice that, for γ=3.2 and $k_{\min}=3$, $k_{0}^{*}=729$, a quite large value, while it decays quickly for increasing γ: For γ=3.5, it is already $k_{0}^{*}=27$. In this regime, the situation is similar to the case γ<3, with practically all active nodes belonging to the DOPGC and $S_{\mathrm{DOP}}\approx N_{a}/N∼k_{a}^{1-\gamma }$. However, one must notice that, even if $k_{a}/k_{c}>1$, this ratio is not very large, as its maximum value is $k_{\min}$, corresponding to $k_{a}=k_{\min}$. Therefore, one never observes the scaling predicted by Eq. (6); as soon as $k_{a}$ is increased, one immediately starts to see the transition to a different regime, where $k_{a}/k_{c}<1$. In this second regime, a giant component still exists, but some active nodes are isolated (not directly connected to other active nodes) and others are nonisolated but form small clusters. The set of all active nodes is therefore composed by three classes:

(1) nonisolated nodes belonging to the DOPGC;
(2) nonisolated nodes belonging to small clusters; and
(3) isolated nodes, which necessarily do not belong to the DOPGC.

**FIG. 2.**
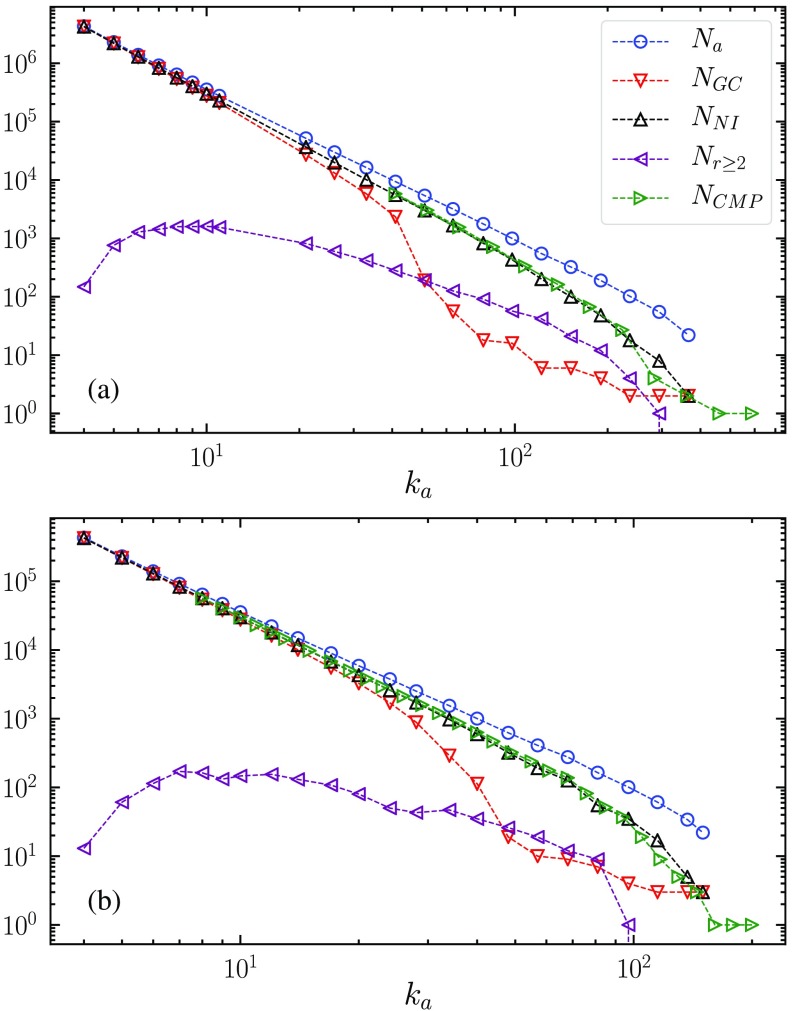
Dependence on $k_{a}$ of the number $N_{a}$ of active nodes, the number $N_{\mathrm{GC}}$ of nodes in the DOP giant component, the number $N_{\mathrm{NI}}$ of nonisolated nodes, the number $N_{r\geq 2}$ of isolated nodes with range r≥2, and the number $N_{\mathrm{CMP}}$ of nodes in the CMP giant component. Results are for power-law networks with γ=3.5, $k_{\min}=3$, and size $N=10^{7}$ (a) and $N=10^{6}$ (b), generated using the uncorrelated configuration model (UCM) [35].

As $k_{a}$ increases, a growing fraction of active nodes passes from the first category to the other two, and the order parameter $S_{\mathrm{DOP}}=N_{\mathrm{GC}}/N$ decreases faster than the fraction of active nodes $N_{a}/N$ (see Fig. 2). At the threshold, the fraction of nonisolated nodes belonging to the DOPGC vanishes.

The calculation of the behavior of $S_{\mathrm{DOP}}$ in this regime and of the transition point is a nontrivial task. It is important to observe that $N_{\mathrm{NI}}$, the number of nonisolated nodes, which upper bounds the number $N_{\mathrm{GC}}$ of nodes belonging to the giant component, keeps decaying with the same exponent even well above the DOP transition (see Fig. 2).

### B. The case of growing r(m) for γ<3

Let us turn now to the more generic case where r(m) grows as a function of m. The range of interaction grows with its mass, so that, if r(m)≥2, clusters of nodes can merge even if not in direct contact. In this case, it is clear that, for a given value of $k_{a}$, the giant component of the DOP process is a subset of the giant component of the full CMP process (CMPGC). Thus, for γ<3, the CMPGC is again given by the whole set of active nodes and has, therefore, a relative size

$$S=\frac{N_{a}}{N}={\left(\frac{k_{a}}{k_{\min}}\right)}^{1-\gamma }.$$ (8)

### C. The case of growing r(m) for γ>3

In this case, for large $k_{a}$, the DOPGC vanishes asymptotically and nonisolated active nodes form DOP clusters of a small size. Still, an extensive CMPGC could be induced by long-range merging of clusters or nodes which cannot be joined in a DOP process, as they are separated by distances larger than 1. Whether these long-range mergings take place or not depends, of course, on the particular choice of the mass m and of the form of the interaction range. Inspired by Ref. [29], here we focus on the case of initial masses equal to node degrees $m_{i}^{\left(0\right)}=k_{i}$ (so that the total mass of a cluster is the sum of the degrees of the active nodes forming it) and of an interaction range of the form $r(m)=m/k_{a}$. This case is a particular case of a generic CMP process with $r(m)=f(m/k_{a})$, where $f(z)=z^{\alpha }$, with α>0, so that active nodes with the smallest degree have a range exactly equal to 1. We defer to a future work a comprehensive analysis of this model for α≠1.

In the present setting, we identify two competing mechanisms leading to the formation of a CMP giant component. The first is an extension of DOP percolation, based on the merging of DOP clusters separated by distances larger than 1. The second involves the buildup of CMP clusters formed by isolated nodes interacting at a large distance. We now discuss the two mechanisms in detail.

#### 1. First mechanism: Extended DOP mechanism

For very small $k_{a}$ close to $k_{\min}$, CMP is clearly equivalent to the first regime for DOP with $S\approx N_{a}/N$. Upon increasing $k_{a}$, above the crossover scale $k_{0}^{*}$, DOP enters the second regime with an increasing presence of isolated nodes and nodes belonging to small DOP clusters. CMP and DOP behaviors start to diverge at this point, because some nodes, even if they are not directly connected to the DOPGC, are at distance 2 from it and, thus, can join the CMPGC if their interaction range is at least 2. In particular, this situation occurs for all small DOP clusters: As their aggregate degree is $k_{\mathrm{agg}}\geq 2k_{a}$, they necessarily have a range of interaction r≥2. For this reason, in this regime all $N_{\mathrm{NI}}$ nonisolated nodes belong to the CMPGC. This result is clearly verified in Fig. 2. Notice that $N_{\mathrm{NI}}/N$ is finite even well beyond the DOP threshold. In this limit, the formation of the CMPGC is still triggered by the largest DOP cluster (that does not percolate). For any value of γ, there are always nodes in the network with $k>k_{c}\gg k_{a}$. They form local clusters with a large interaction range that progressively incorporate other small clusters giving rise to a CMPGC, even if no DOPGC is present. To calculate $N_{\mathrm{NI}}$, we consider the probability that an active node of degree k has at least one neighboring active node:

$$P_{\mathrm{NI}}(k)=1-(1-P_{a}{)}^{k}\approx 1-e^{-k/k_{c}}.$$ (9)

The total fraction $N_{\mathrm{NI}}/N$ of nonisolated active nodes in a power-law distributed network is then

$$\frac{N_{\mathrm{NI}}}{N}=\int_{k_{a}}^{\infty }dk^{\prime }P(k^{\prime })P_{\mathrm{NI}}(k^{\prime })\;=(\gamma -1)k_{\min}^{\gamma -1}\left[\frac{k_{a}^{1-\gamma }}{\gamma -1}-k_{c}^{1-\gamma }\Gamma \left(1-\gamma ,\frac{k_{a}}{k_{c}}\right)\right],$$ (10)

where Γ(a,z) is the incomplete Gamma function [36].

In this second regime, not only small DOP clusters, but also isolated active nodes can join the CMPGC, provided they have degree $k\geq 2k_{a}$ so that their range is r≥2. We denote their number as $N_{r\geq 2}$. The total fraction of isolated nodes with range r≥2 is

$$\frac{N_{r\geq 2}}{N}=\int_{2k_{a}}^{\infty }dk^{\prime }P(k^{\prime })[1-P_{\mathrm{NI}}(k^{\prime })]$$ (11)

$$=(\gamma -1)k_{\min}^{\gamma -1}k_{c}^{1-\gamma }\Gamma \left(1-\gamma ,\frac{2k_{a}}{k_{c}}\right).$$ (12)

Overall, the CMP order parameter in this regime is, therefore,

$$S_{1}\approx \frac{N_{\mathrm{NI}}}{N}+\frac{N_{r\geq 2}}{N}.$$ (13)

For $k_{a}\to k_{\min}$, one has $k_{a}>k_{c}$, and the first contribution in Eq. (13) is larger than the second, for any γ. For large $k_{a}$, instead, one can expand the Γ functions for small $k_{a}/k_{c}$, finding

$$\frac{N_{\mathrm{NI}}}{N}={\left(\frac{k_{a}}{k_{\min}}\right)}^{1-\gamma }\left[\frac{\gamma -1}{\gamma -2}\frac{k_{a}}{k_{c}}\right]∼k_{a}^{2(2-\gamma )}$$ (14)

and

$$\frac{N_{r\geq 2}}{N}={\left(\frac{2k_{a}}{k_{\min}}\right)}^{1-\gamma }\left[1-\frac{\gamma -1}{\gamma -2}\frac{2k_{a}}{k_{c}}\right]∼k_{a}^{1-\gamma }.$$ (15)

The exponent of $N_{\mathrm{NI}}$ is, in absolute value, larger than the one of $N_{r\geq 2}$; hence, the first contribution dominates up to a crossover scale

$$k_{1}^{*}={\left[\frac{\left(\gamma -2\right)}{\left(\gamma -1\right)}\frac{2^{\left(1-\gamma \right)}}{\left(1+2^{2-\gamma }\right)}k_{\min}^{\left(2-\gamma \right)}\right]}^{1/(3-\gamma )}.$$ (16)

The conclusion of this line of reasoning is that for $k_{a}\ll k_{1}^{*}$ the size of the CMPGC decays as

$$S_{1}\approx \frac{N_{\mathrm{NI}}}{N}∼k_{a}^{2(2-\gamma )}$$ (17)

followed by a crossover to $S_{1}\approx (N_{r\geq 2}/N)∼k_{a}^{1-\gamma }$. The crossover scale $k_{1}^{*}$ decreases rapidly with γ, but, since the maximum degree in a network grows as $N^{1/(\gamma -1)}$, the minimum network size necessary to have a sufficiently large maximum degree $k_{\max}=k_{1}^{*}$ is always larger than $N\approx 4.2\times 10^{5}$ (the minimum occurring for γ≈5 for $k_{\min}=3$). Hence, it should be possible to observe the crossover on large networks (although $k_{\max}$ grows very slowly with N; hence, one needs networks of a size much larger than $10^{5}$ nodes to have a still limited range of $k_{a}$ values). As a matter of fact, we do not observe such a crossover.

**FIG. 3.**
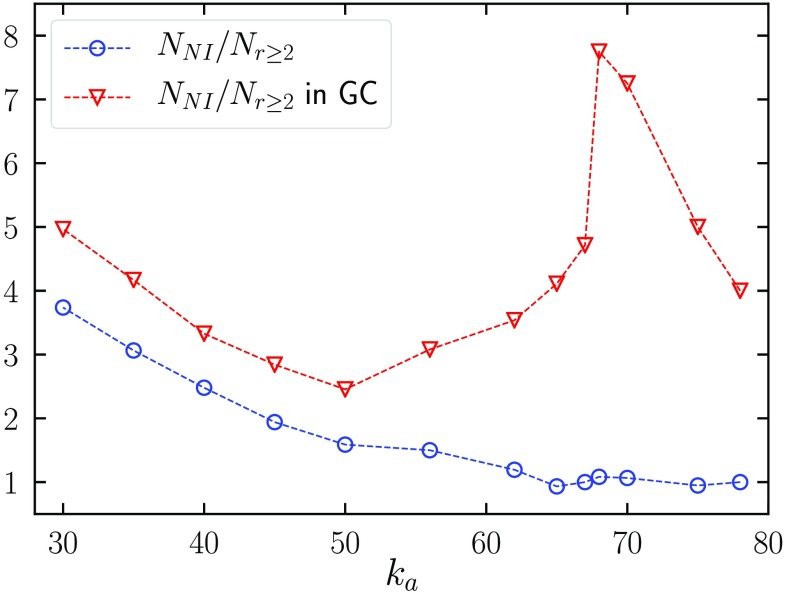
Ratio $N_{\mathrm{NI}}/N_{r\geq 2}$ between the two terms in Eq. (13) evaluated on UCM networks with γ=4, $k_{\min}=3$, and size $N=10^{7}$, computed over all nodes, and restricted to nodes belonging to the CMPGC.

This result happens because, as $k_{a}$ grows, the extended DOP mechanism becomes less and less effective. DOP clusters become smaller and smaller, and the distances among them (and between isolated active nodes and them) increase: It is no longer sufficient to have r=2 to join the CMP giant component. For even larger $k_{a}$, it is not even sufficient to have r=3 or r=4 and so on. This effect suppresses both terms in Eq. (13), but the second term is most affected, as can be seen in Fig. 3, where we compare the ratio of the first and second terms in Eq. (13) (which becomes 1 at the crossover scale $k_{1}^{*}$) and the same ratio restricted to nodes belonging to the CMPGC. We observe that the latter is always larger than the former and does not seem to go to 1 for large $k_{a}$. This result implies that, in practice, $S_{1}$ behaves as predicted by Eq. (17) even for values of $k_{a}$ larger than the crossover scale $k_{1}^{*}$ estimated in Eq. (16).

A more important consequence of the asymptotic ineffectiveness of the extended DOP mechanism is that it cannot work for arbitrarily large $k_{a}$. A different mechanism governs the formation of the CMPGC in the limit $k_{a}\to \infty$.

#### 2. Second mechanism: Merging of distant isolated nodes

Nodes with degree $k_{a}\leq k\ll k_{c}$ have on average a very small number of active nearest neighbors, as $kP_{a}=k/k_{c}\ll 1$. Hence, they are typically isolated. However, if k is large enough, they may still have a large interaction range and may merge with other distant nodes. To analyze this process in detail, let us denote as d(k) the mean distance between a node of degree k and the closest node of degree at least k. In the limit of a large network size, this distance is (see Appendix B for an analytical derivation)

$$d(k)\approx 1+\frac{\gamma -3}{\ln(\kappa )}\ln\left(\frac{k}{k_{\min}}\right),$$ (18)

where $\kappa =\langle k^{2}\rangle /\langle k\rangle -1$ is the network branching factor. Since the interaction range of a node grows linearly with its degree k, it grows faster than the distance to its closest peer. Hence, there exists a degree $k_{x}$ such that

$$r(k_{x})=d(k_{x})$$ (19)

and, for any $k>k_{x}$, r(k)>d(k). As a consequence, nodes with $k>k_{x}$ have an interaction range larger (on average) than their mutual topological distance. They can thus merge in pairs with an even larger interaction range, and the process repeats itself, leading to the formation of a CMPGC, comprising all nodes with a degree larger than $k_{x}$. If we write $k_{x}$ in the form $k_{x}=\omega k_{a}$, the condition (19) implies $\omega =d(\omega k_{a})$, which, inserting the explicit expression of d(k), becomes

$$\omega \approx 1+\frac{\gamma -3}{\ln(\kappa )}\ln\left(\frac{\omega k_{a}}{k_{\min}}\right).$$ (20)

Neglecting constants and terms of the order of $\ln[\ln(k_{a})]$, the size of the giant component according to this mechanism scales then as

$$S_{2}\approx {\left(\frac{k_{x}}{k_{\min}}\right)}^{1-\gamma }=\omega^{1-\gamma }{\left(\frac{k_{a}}{k_{\min}}\right)}^{1-\gamma }$$ (21)

$$={\left[\frac{\gamma -3}{\ln(\kappa )}\ln\left(\frac{k_{a}}{k_{\min}}\right)\right]}^{\left(1-\gamma \right)}{\left(\frac{k_{a}}{k_{\min}}\right)}^{1-\gamma },$$ (22)

showing, thus, a power-law decay times a logarithmic correction.

**FIG. 4.**
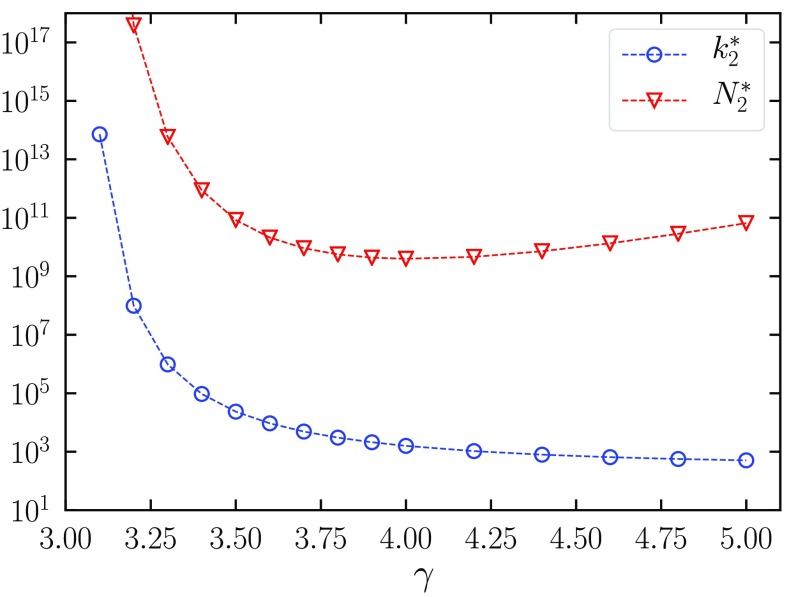
Values of the crossover degree $k_{2}^{*}$ and the minimal network size $N_{2}^{*}=k_{2}^{*(\gamma -1)}$ as a function of γ. In order to observe the crossover, networks of size $N\gg N_{2}^{*}$ should be considered.

In absolute value, the leading exponent in $S_{2}$ is smaller than the exponent in $S_{1}$. Therefore, we expect this second mechanism to dominate asymptotically, but after a crossover preceded by a scaling regime where the size of the CMPGC is given by Eq. (17). The position $k_{2}^{*}$ of the crossover is estimated by numerically solving the equation $S_{1}(k_{2}^{*})=S_{2}(k_{2}^{*})$. Figure 4 shows how this quantity decreases with the exponent γ. However, in order to observe such a crossover, one must consider networks much larger than $N_{2}^{*}=k_{2}^{*(\gamma -1)}$. These values are huge for any γ (much larger than $10^{9}$ nodes in the best case), leading to the conclusion that only the first regime can be observed in currently feasible simulations.

The present analysis can be extended also to the case of networks with a stretched exponential degree distribution, predicting an asymptotic stretched exponential dependence of S on $k_{a}$. See Appendix C for details.

## IV. FINITE-SIZE EFFECTS

So far, we consider infinitely large networks, thus assuming that all degree classes, up to infinity, exist. When the network size is finite, only degrees up to the maximum value $k_{\max}(N)$, growing as $N^{1/(\gamma -1)}$, are present [37]. The CMP behavior for the infinite network (i.e., there is a CMPGC for any $k_{a}$) holds as long as $k_{\max}$ is larger than the degree scale involved in the formation of the CMPGC.

For γ<3, it is sufficient to have active nodes for observing an extensive CMPGC. Hence, the only finite-size effect trivially appears for $k_{a}>k_{\max}(N)$: In such a case, there are no more active nodes in the system and S≈0. The finite-size effective threshold is $k_{a}^{c}=k_{\max}(N)$.

On the contrary, for γ>3, finite-size effects are less trivial. The presence of active nodes is not sufficient to give rise to a CMPGC. One needs the presence of nodes with $k>k_{c}$ (first mechanism) or $k>k_{x}$ (second mechanism). Notice that, since $k_{c}$ grows as a power of $k_{a}$ with an exponent larger than 1, while $k_{x}$ grows logarithmically, asymptotically $k_{c}\gg k_{x}$. Different scalings of the finite-size effective threshold are possible, depending on whether the maximum degree $k_{\max}(N)$ is larger or smaller than the crossover degree $k_{2}^{*}$.

If $k_{\max}(N)>k_{2}^{*}$, finite-size effects appear during the regime where the formation of the CMPGC is governed by the second mechanism. In this case, the asymptotic behavior $S\approx S_{2}$ ends (i.e., S≈0) when the relevant degree scale $k_{x}$ (growing with $k_{a}$) becomes larger than $k_{\max}(N)$. In such a case, there are active nodes in the system, but neither of the two mechanisms for the formation of the giant component is at work. The effective threshold in this case is given by the condition $k_{x}=k_{\max}(N)$, implying asymptotically

$$k_{a}^{c}∼\frac{\ln(\kappa )}{\gamma -3}\frac{k_{\max}(N)}{\ln[k_{\max}(N)]}.$$ (23)

If, instead, $k_{\max}(N)<k_{2}^{*}$, finite-size effects start to appear already during the preasymptotic regime where the first mechanism rules. As soon as $k_{c}>k_{\max}(N)$, the behavior $S\approx S_{1}$ ends. The effective threshold is thus given by the condition $k_{c}=k_{\max}(N)$, implying

$$k_{a}^{c}=k_{\min}k_{\max}^{1/(\gamma -2)}.$$ (24)

Notice that after this effective threshold the order parameter does not go to S≈0, as there is still an interval of $k_{a}$ values such that $k_{x}<k_{\max}(N)<k_{c}$. In this regime, the first mechanism is no longer operative; still, the second is at work, but since $N<N_{2}^{*}$, it cannot lead to a macroscopic giant component.

## V. NUMERICAL TEST

**FIG. 5.**
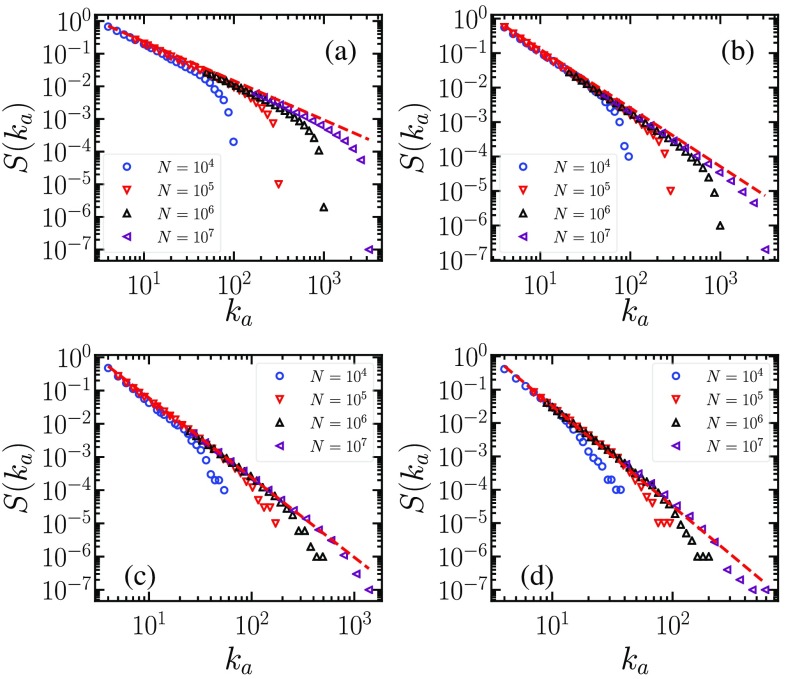
Fraction S of nodes in the largest CMP component as a function of $k_{a}$ for various γ values: γ=2.2 (a), γ=2.7 (b), γ=3.2 (c), and γ=3.5 (d). In all cases, $k_{\min}=3$. Symbols represent numerical results for various network sizes. Dashed lines are theoretical predictions from Eqs. (8) [(a),(b)] and (17) [(c),(d)].

We test the correctness of the scaling analysis performed in the previous sections by means of numerical simulations of the CMP process with $r(m)=m/k_{a}$. In Figs. 5(a) and 5(b), we report, as a function of $k_{a}$, the fraction S of nodes in the largest CMP cluster for γ<3 on uncorrelated configuration model networks (UCM) [35] of various sizes. The plot shows the presence of a CMPGC, including a fraction of active nodes independent of the system size N. The scaling of S with $k_{a}$ is in excellent agreement with the prediction of Eq. (8). Finite-size effects are also apparent and perfectly agree with the prediction formulated above: The effective threshold occurs for $k_{a}=k_{\max}(N)=N^{1/2}$. Increasing the network size, the effective threshold diverges: Asymptotically, there is a giant component for any $k_{a}>0$.

For γ>3, we consider a hard cutoff $M=N^{1/(\gamma -1)}$ for the degree sequence generated in the UCM model, in order to avoid the possible appearance of outliers having a degree much larger than the average $k_{\max}$ [38]. Figures 5(c) and 5(d) show that also in this case the fraction of active nodes in the CMPGC is extensive, and its dependence on $k_{a}$ is well described by Eq. (17). This result confirms the depicted scenario about the formation of an extensive CMPGC and points out that for the sizes considered only the preasymptotic scaling regime $S_{1}$ is observed, while, as expected, we do not see any trace of the asymptotic behavior (for an infinite network) $S=S_{2}\approx k_{a}^{1-\gamma }$.

Concerning finite-size effects, for γ>3 as only the first scaling regime is observed, the condition setting the effective threshold is Eq. (24). A direct numerical verification of it for CMP is very hard, as practically all nonisolated nodes are part of the CMPGC, and finite clusters (upon which methods to determine the position of the threshold are based) are extremely rare. An indirect numerical verification is provided below in the application to the SIS model. The observation of the effective threshold associated to the second mechanism [Eq. (23)] is impossible in practice, as it requires huge networks of a size larger than $N_{2}^{*}$.

The conclusion of our analysis is that, in different manners depending on whether γ<3 or γ>3, a CMP giant component is present in infinite networks for any value of $k_{a}$. The threshold for this class of CMP processes is infinite for any value of γ.

## VI. APPLICATION TO SIS EPIDEMIC SPREADING

The theoretical picture presented in the previous sections can be applied to the CMP process associated to SIS dynamics, which is an instance of this class with $k_{a}=a/\lambda^{2}\ln(1/\lambda )$, initial mass equal to the degree, and $r(m)=m/k_{a}$; see Appendix A. This application has mainly the goal of investigating the properties of the SIS epidemic transition for γ>3. We notice that the relation between CMP and SIS depends on the parameter a relating $k_{a}$ with λ, whose value, either a=1 or a=4, is not theoretically determined. In our application of CMP to SIS, we choose to compare with both values.

### A. Scaling of the CMP giant component

The scaling of S with λ for γ<3 is obtained by inserting the expression for $k_{a}$ as a function of λ into Eq. (8), obtaining

$$S=\frac{N_{a}}{N}∼k_{a}^{1-\gamma }∼\lambda^{2(\gamma -1)}\ln^{1-\gamma }\left(\frac{1}{\lambda }\right).$$ (25)

Thus, the approach predicts the existence of a CMPGC for any value of λ>0. Notice, however, that, while it is possible to define a CMP process associated to SIS dynamics for any value of γ, the SIS epidemic transition for γ<5/2 is due to a mechanism different from the mutual reinfection of distant hubs [21]: Hence, SIS critical properties have nothing to do with those of CMP in this case. Moreover, the connection between the scaling of S and the scaling of the SIS prevalence is not trivial in this case; hence, we cannot derive from CMP any prediction on the latter even for 5/2<γ<3.

For γ>3, the fraction of active nodes in the CMPGC is extensive, and its preasymptotic dependence on λ is obtained by plugging the expression for $k_{a}$ into Eq. (17):

$$S_{1}=\frac{N_{\mathrm{NI}}}{N}∼k_{a}^{2(2-\gamma )}∼\lambda^{4(\gamma -2)}\ln^{2(2-\gamma )}\left(\frac{1}{\lambda }\right).$$ (26)

We can also calculate the asymptotic scaling of the CMPGC, by plugging the expression for $k_{a}$ into the expression of the scaling of the CMP giant component in the second regime, Eq. (22), obtaining

$$S_{2}∼\ln^{1-\gamma }(k_{a})k_{a}^{1-\gamma }∼\lambda^{2(\gamma -1)}\ln^{2(1-\gamma )}\left(\frac{1}{\lambda }\right).$$ (27)

We recall, however, that this scaling occurs only for exceedingly large values of $k_{a}$ (i.e., values of λ exceedingly small), so that it cannot be observed in present simulations.

### B. Finite-size epidemic threshold

For γ>3, as only the first scaling regime is observed, the condition setting the effective threshold is $k_{\max}(N)=k_{c}(\lambda )$, i.e.,

$$\frac{a}{\lambda_{c}^{2}}\ln\left(\frac{1}{\lambda_{c}^{2}}\right)=k_{\min}k_{\max}^{1/(\gamma -2)}.$$ (28)

This result translates (apart from logarithmic corrections) into

$$\lambda_{c}(N)=(a/k_{\min}{)}^{1/2}k_{\max}^{-1/[2(\gamma -2)]}.$$ (29)

**FIG. 6.**
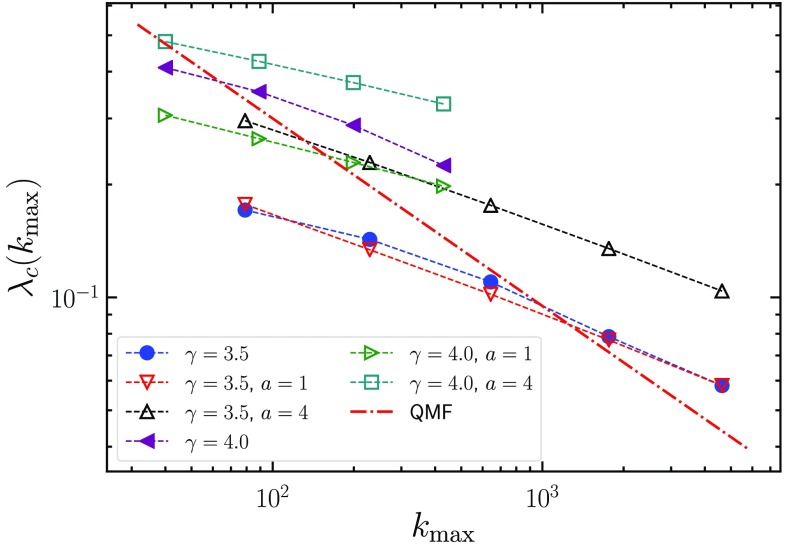
Comparison between the theoretical finite-size threshold (for two different values of a) (hollow symbols) and direct numerical simulations (full symbols) of the SIS process in UCM networks with degree exponent γ=3.5 ($k_{\min}=3$) and γ=4 ($k_{\min}=2$) [24]. The epidemic threshold is determined by means of the lifespan method [24]. The dashed red line is proportional to the prediction $1/\sqrt{k_{\max}}$ of the QMF theory. The values of $k_{\max}$ correspond to sizes ranging from $N=10^{4}$ to $N=10^{8}$ for γ=3.5 and from $N=10^{4}$ to $N=10^{7}$ for γ=4.

For $k_{\max}^{-1/2}<\lambda <\lambda_{c}(N)$, there are active hubs in the system, but they do not give rise to a CMPGC. Hence, $\lambda_{c}(N)$ can be identified with the effective size-dependent epidemic threshold. Equation (29) is very interesting, as it shows that the effective threshold does not vanish as $k_{\max}^{-1/2}$, as predicted by the QMF theory, but *more slowly*, with an exponent that is reduced as γ is increased. The prediction of Eq. (28) is compared in Fig. 6 with SIS numerical results of Ref. [24], displaying a good agreement and thus clarifying a long-standing open issue. For reference, we also report the scaling predicted by the QMF theory, which patently disagrees with numerical results.

Notice, however, that this result is not the final asymptotic behavior of $\lambda_{c}(N)$. For much larger networks, it could be possible (at least in principle) to reach values of $k_{a}$ larger than the crossover value $k_{2}^{*}$. In such a case, the decay of the effective threshold would be given by the condition $k_{x}=k_{\max}(N)$, that, from Eq. (20), leads to

$$\lambda_{c}(N)=\omega^{1/2}k_{\max}^{-1/2}∼\ln(k_{\max})k_{\max}^{-1/2}.$$ (30)

In this way, we recover the asymptotic scaling of the effective threshold recently derived by Huang and Durrett [27].

In Appendix D, we show that the CMP approach provides the correct effective finite-size threshold also in the case of stretched exponential degree distributions.

### C. SIS prevalence as a function of λ

Above the size-dependent effective threshold, there is a backbone of active nodes which sustain an endemic state by reinfecting each other. In an infinite network, when the CMP giant component is formed by distant, mutually interacting hubs (second regime), we can estimate the value of the prevalence (average density of infected nodes) for small λ using the following argument. All actives nodes with a degree larger than $k_{x}=\omega k_{a}$ participate in the CMPGC. Each one of these active nodes of degree k infects a number of other nodes of order λk. Since hubs are distant, these clusters of infected nodes do not overlap; hence, the total prevalence in the system is expected to be [23]

$$\rho ∼\int_{k_{x}}^{\infty }dk\lambda kP(k)∼\lambda (\omega k_{a}{)}^{2-\gamma }.$$ (31)

Substituting the values of ω and $k_{a}$ into Eq. (31) leads to

$$\rho (\lambda )∼\lambda^{2\gamma -3}{\left[\ln(1/\lambda )\right]}^{2(2-\gamma )},$$ (32)

in agreement with the exact mathematical results of Mountford, Valesin, and Yao [28]. As discussed above, this prediction is, however, impossible to verify numerically, because the onset of the asymptotic regime could be seen only for exceedingly large networks, which explains the mismatch between the theory of Mountford, Valesin, and Yao and numerical results. In doable simulations of the SIS model, the small λ regime that can be observed is the preasymptotic regime $S_{1}$ for the corresponding CMP process. In such a regime, since hubs are not well separated, it is not possible to assume that each of them independently infects a number of neighbors of the order of λk. The derivation of the exponent characterizing the SIS prevalence singularity in this preasymptotic (but long) regime remains an interesting open question for future research.

## VII. DISCUSSION

In this paper, we consider a long-range percolation process, the cumulative merging percolation, exhibiting a rich phenomenology that we have uncovered developing an appropriate scaling theory. While we mainly focus on particular forms of the model inspired by the analysis of SIS process [29], more complex scenarios can be obtained by changing the functional form of the interaction range r(m) or the activation probability $p_{i}$ and by considering a more complicated mass merging function $m_{\alpha +\beta }=\;g(m_{\alpha },m_{\beta })$. In this sense, we expect other types of percolation transitions to arise as these features are changed. For example, if r(m) saturates to a finite value when m diverges, the arguments presented above imply the presence of a finite threshold for γ>3 as for the DOP process. The investigation of the general phenomenology of the CMP process and of its connections with other models is a promising avenue for future research.

Concerning the application of CMP to SIS dynamics, our results clarify how the mutual reinfection mechanism among distant hubs, underlying the epidemic transition for γ>3 [24], takes place. This clarification closes the last gap in our understanding of the SIS dynamics and leads to a complete and consistent physical picture that we sketch here.

**FIG. 7.**
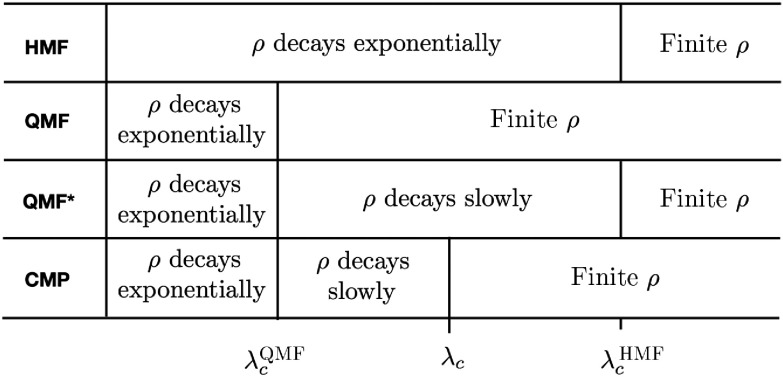
Behavior of SIS prevalence ρ according to the different approaches. QMF* stands for the QMF theory as reinterpreted in Refs. [22,23].

The original heterogeneous-mean-field theory (HMF) [15,16], based on an annealed network approximation [2], predicts an epidemic threshold given $\lambda_{c}^{\mathrm{HMF}}=\langle k\rangle /\langle k^{2}\rangle$ and thus finite in the limit of infinite-size networks for γ>3 (see Fig. 7). Below $\lambda_{c}^{\mathrm{HMF}}$, this theory predicts a density of infected individuals ρ(t) decaying exponentially to zero. For $\lambda >\lambda_{c}^{\mathrm{HMF}}$, the HMF predicts a finite ρ in the steady state.

The quenched-mean-field theory (QMF or NIMFA) [17–19] predicts the same scenario, a transition separating an active steady state with finite prevalence from an absorbing phase where the prevalence decays exponentially to zero. The difference with respect to the HMF is in the value of the threshold, $\lambda_{c}^{\mathrm{QMF}}=1/\Lambda_{M}$, where $\Lambda_{M}$ is the largest eigenvalue of the adjacency matrix, which vanishes as N diverges, for any γ. The value of the QMF threshold is the minimum value of λ such that the star graph composed by the largest hub and its direct neighbors is able to independently sustain long-lasting activity [21]. Correspondingly, the principal eigenvector for γ>5/2 is localized around the largest hub [22]. Also, the other leading eigenvalues of the adjacency matrix are associated to eigenvectors localized on each of the hubs of the network. In the thermodynamic limit, for any given value of λ, each large hub with degree $k>1/\lambda^{2}$ sustains long-lasting activity together with its direct neighbors, yielding an overall finite density of infected individuals that can be estimated [23] as $\rho ∼\lambda^{2\gamma -3}$.

However, as pointed out in Refs. [22,23], this scenario cannot really hold for SIS dynamics. In the QMF theory, there are no stochastic fluctuations, and activity in a star graph composed by k+1 nodes persists forever if $\lambda >1/\sqrt{k}$. In SIS dynamics, instead, activity survives, in a star graph made of k+1 nodes, only for a time of the order of $\exp(\lambda^{2}k/a)$. Hence, if star graphs are independent, the overall activity does not survive for a time scaling exponentially with the system size N. In other words, there is some activity surviving for some time but not a truly steady active state. In the interval $\lambda_{c}^{Q\mathrm{MF}}<\lambda <\lambda_{c}^{\mathrm{HMF}}$, one should expect a Griffiths-like phase, with a slow decay of ρ(t) [23], due to the convolution of contributions from a decreasing number of still active individual hubs. Only above a *finite* threshold, approximately equal to $\lambda_{c}^{\mathrm{HMF}}$, corresponding to the inverse of the first eigenvalue associated to a delocalized eigenvector [22], is a truly active steady state expected (see Fig. 7). This scenario is consistent, but at odds with numerical simulations [24] and exact mathematical results [39,40], which find an active steady state for any value of λ>0 in the thermodynamic limit.

One possible way to reconcile these findings was explored by Lee, Shim, and Noh [23]. If large hubs are in mutual direct contact (i.e., they form an extensive connected cluster), should activity spontaneously disappear in one of them, neighboring hubs would be able to reinfect it; these mutual reinfections would lead to a survival time exponential in N, i.e., a truly active steady state. Unfortunately, the study of degree-ordered-percolation reveals that such an extensive cluster of large hubs always exists only for γ<3. For γ>3, the DOP threshold is finite: The largest hubs are separated and do not form an extensive cluster [23].

Our results go beyond those of Lee, Shim, and Noh and clarify what is missing in previous approaches: The activity triggered by hubs extends beyond nearest neighbors, up to a scale that grows with λ, so that hubs can interact even if they are not in direct contact. This long-range interaction gives rise, above a critical value $\lambda_{c}(N)$, to an extensive CMP percolating cluster of active nodes able to reinfect each other at distance and, thus, giving a veritable steady state with finite prevalence ρ. The threshold $\lambda_{c}(N)$ is intermediate between $\lambda_{c}^{\mathrm{QMF}}$ and $\lambda_{c}^{\mathrm{HMF}}$ and vanishes as a function of N (at odds with $\lambda_{c}^{\mathrm{HMF}}$) but more slowly than $\lambda_{c}^{\mathrm{QMF}}$. Considering finite networks, while for $\lambda_{c}(N)<\lambda <\lambda_{c}^{\mathrm{HMF}}$ a CMP percolating cluster exists and prevalence is finite, for $\lambda_{c}^{\mathrm{QMF}}<\lambda <\lambda_{c}$ only small nonpercolating CMP clusters are present. In this case, each of them decays independently, and, thus, a Griffiths-like phase, characterized by ρ(t) slowly decaying to zero, is expected (Fig. 7). A numerical validation of this prediction, which is difficult as both interval bounds vanish with the system size, remains a challenge for future numerical studies.

The consideration of long-range effects is the crucial ingredient in our analysis that makes a qualitative difference with previous approaches. While the QMF theory neglects correlations among the dynamical state of neighbors, other theories [41–43] take some correlations into account, but, since they consider only neighbors in a short range, they cannot capture the long-range percolative nature of the SIS epidemic transition for γ>3.

Our work puts in proper place the different theories presented in recent years to explain the behavior of the SIS model in heterogeneous networks, showing, in particular, the limit in which exact mathematical results are expected to be observed, putting thus an end to the long debate on this subject. On the other hand, it opens new perspectives, as it proposes the cumulative merging of distant clusters as a very generic phenomenon which may originate nontrivial types of percolation phenomena in networks.

## Figures and Tables

**FIG. 8.**
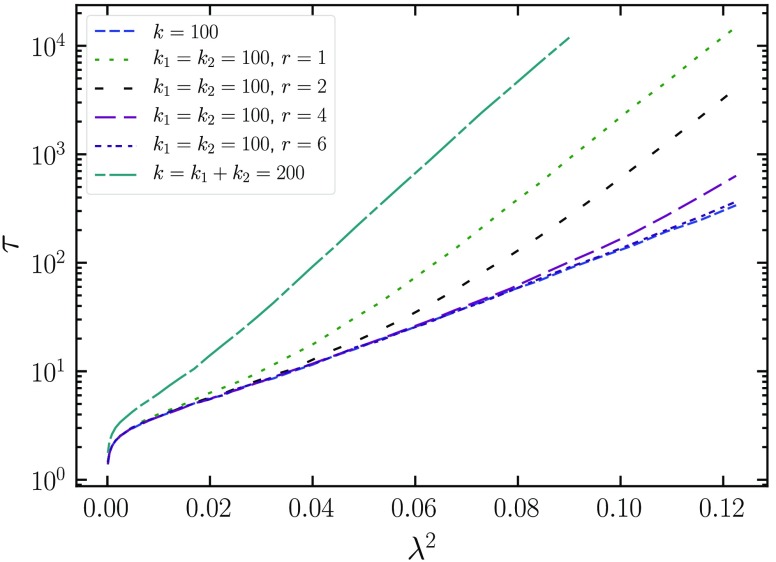
Average survival time τ of a SIS epidemic on two star graphs of size $k_{1}=k_{2}=k$, connected by a line of r-1 intermediate nodes, starting with only the hub of one of them in the infected state. We compare with the result for single stars of size k and 2k. As we can see, for sufficiently small values of r (distance between the hubs), the survival time of the connected stars of size k scales with $\lambda^{2}$ as a single star of size 2k.

**FIG. 9.**
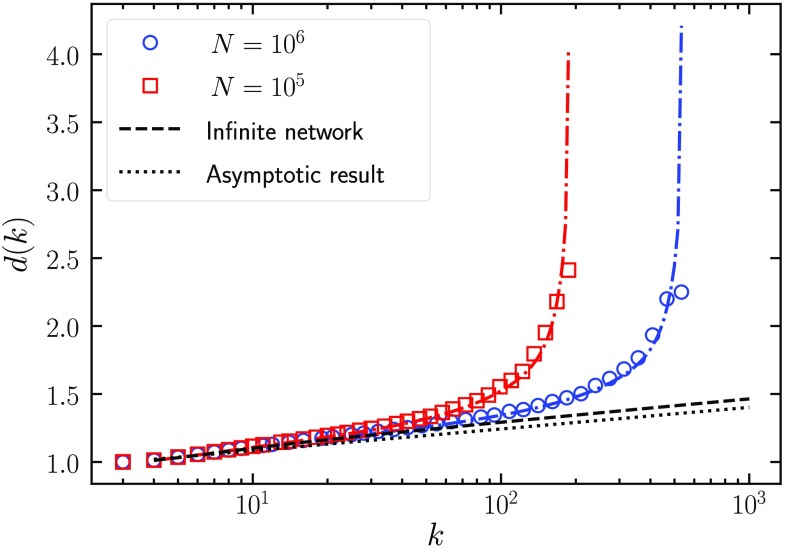
Average distance between a node of degree k and the closest node of a degree at least k in power-law networks with degree exponent γ=3.2. Symbols represent numerical simulations over networks of different sizes, averaged over $10^{4}$ independent network samples. The dashed line represents the numerical evaluation of the summation Eq. (B6) in the infinite network limit. Dot-dashed lines represent results of the summation for finite networks of the corresponding size N. The dotted line corresponds to the asymptotic expression Eq. (B9).

## APPENDIX A: CONNECTION BETWEEN CMP AND SIS

The SIS model, often called the contact process in the community of applied probabilists, is defined as follows: Individuals can be in one of two states, either susceptible or infected. Susceptible individuals become infected by contact with infected individuals, at a rate equal to the number of infected contacts times a given spreading rate β. Infected individuals, on the other hand, become spontaneously healthy again at a rate μ. The ratio λ=β/μ is the control parameter for the model, which experiences a transition between a healthy and an endemic (infected) steady state when λ crosses an epidemic threshold $\lambda_{c}$. In power-law distributed networks, for γ>5/2 the epidemic transition is triggered by nodes with a large number k of neighbors (hubs). Each of these hubs together with its direct neighbors (leaves) forms a star graph, which in isolation is able to sustain the survival of the epidemic for a long time, $\tau (k)∼\exp(\lambda^{2}k/4)$ [24], provided λ is larger than $\lambda_{c}(k)=1/\sqrt{k}$. During this long time interval, even if the hub recovers from the infection, it is promptly reinfected by one of its neighbors and can in its turn reinfect other leaves when they recover. After a typical time τ, a fluctuation takes the star graph formed by a hub and its nearest neighbors to the absorbing state.

Since the star graph is not isolated in the network, it can propagate activity to other nodes. It is possible to estimate [24] the average time it takes for an infected node to infect for the first time a node at distance r in the limit of small λ:

$$T(r)∼e^{r\ln(1/\lambda )}.$$ (A1)

By equating τ and T(r), it is possible to estimate the “range of interaction” of a hub of degree k, i.e., the maximum distance at which a star surrounding an active hub is able to propagate the infection before spontaneously recovering:

$$r(k)=\frac{\lambda^{2}k}{4\ln(1/\lambda )}=\frac{k}{k_{a}},$$ (A2)

where we define

$$k_{a}=4\frac{1}{\lambda^{2}}\ln\left(\frac{1}{\lambda }\right).$$ (A3)

Consider now another hub, of degree $k^{\prime }$ at a distance $r_{0}$ from the first. If $r_{0}<r(k)$, the second hub is infected by the first and it is able to stay infected (together with its direct neighbors) for a time $\tau (k^{\prime })$. During this time interval, it spreads the infection up to a distance $r(k^{\prime })$. If $r_{0}>r(k^{\prime })$, this means that the second hub is not able to reinfect the first, should it fall into the absorbing state. Conversely, if $r_{0}<r(k^{\prime })$, even if the first hub recovers, it is reinfected by the second. In this way, the two distant hubs form a coupled system such that if one hub recovers, the other is able to reinfect it before recovering in its turn. For the infection to die out in the system of the two hubs, they must recover almost simultaneously [29]. These concurrent recoveries happen after a time of the order of $\tau (k)\tau (k^{\prime })∼\;\exp[\lambda^{2}(k+k^{\prime })]$; see Fig. 8.

Hence, the combined set of hubs are able to infect nodes at an increased range of interaction $r(k+k^{\prime })$. It is then clear that SIS dynamics can be seen as an instance of the cumulative merging process, with active nodes those with $k\geq k_{a}=4/\lambda^{2}\ln(1/\lambda )$, initial masses equal to node degrees $m_{i}^{\left(0\right)}=k_{i}$, and range of interaction given by $r(m)=m/k_{a}$. Notice that the factor of 4 in the expression for $k_{a}$ is the consequence of the choice $\tau (k)∼\exp(\lambda^{2}k/4)$. Alternative treatments [27,44] give that star graphs are active for $k>k_{a}=1/\lambda^{2}\ln(1/\lambda )$. In the comparison of the CMP approach to SIS with numerical simulations, we consider both expressions.

## APPENDIX B: AVERAGE DISTANCE BETWEEN A NODE OF DEGREE k AND THE CLOSEST NODE OF DEGREE AT LEAST k

We can estimate the average distance d(k) between a node of degree k and the nearest node of degree larger than or equal to k within a treelike approximation [2]. For random uncorrelated networks, the probability that a link points to a node of degree $k^{\prime }$ is $k^{\prime }P(k^{\prime })/\langle k\rangle$. Arriving at this node, there are $k^{\prime }-1$ possible outgoing edges (excluding the one used to arrive to node $k^{\prime }$). The average number of outgoing edges (the so-called branching factor) is, thus,

$$\kappa =\int_{k_{\min}}^{\infty }(k^{\prime }-1)\frac{k^{\prime }P(k^{\prime })}{\left\langle k\right\rangle }dk^{\prime }=\frac{\left\langle k^{2}\right\rangle }{\left\langle k\right\rangle }-1,$$ (B1)

that is, a finite number for power-law networks with γ>3. From this branching ratio, we estimate the average number of nodes at distance n as $N_{n}=k\kappa^{n-1}$, assuming the tree approximation.

A node of degree k has k neighbors. It is connected at distance d=1 to a node of degree not less than k if at least one of these neighbors has a degree larger than or equal to k. The probability of this event is

$$P_{>}(k)=\int_{k}^{\infty }\frac{k^{\prime }P(k^{\prime })}{\left\langle k\right\rangle }dk^{\prime }={\left(\frac{k}{k_{\min}}\right)}^{2-\gamma }.$$ (B2)

Therefore, the probability that the distance at the nearest neighbors with a degree larger than or equal to k is equal to d=1 is

$$P(d=1)=1-{\left[1-P_{>}(k)\right]}^{k}=1-{\left[P_{<}(k)\right]}^{k},$$ (B3)

where $P_{<}(k)=1-P_{>}(k)$ is the probability that a nearest neighbor of a node has a degree smaller than k.

The nearest neighbor with a degree not less than k is at distance d=2 if there are no such neighbors at distance d=1, and at least one of the neighbors at distance d=2, in number kκ, has a degree not less than k, which happens with probability

$$P(d=2)={\left[P_{<}(k)\right]}^{k}\left\{1-{\left[P_{<}(k)\right]}^{k\kappa }\right\}.$$ (B4)

By induction, we can see that the nearest neighbor of a degree not less that k is at distance d=n corresponds to not observing one at any distance smaller than n and having at least one at a distance equal to n, which happens with probability

$$P(d=n)={\left[P_{<}(k)\right]}^{k}{\left[P_{<}(k)\right]}^{k\kappa }{\left[P_{<}(k)\right]}^{k\kappa^{2}}\ldots {\left[P_{<}(k)\right]}^{k\kappa^{n-2}}\left\{1-{\left[P_{<}(k)\right]}^{k\kappa^{n-1}}\right\}\;={\left[P_{<}(k)\right]}^{\overset{n-2}{\underset{r=0}{\sum }}k\kappa^{r}}-{\left[P_{<}(k)\right]}^{\overset{n-1}{\underset{r=0}{\sum }}k\kappa^{r}}\;={\left[P_{<}(k)\right]}^{k(\kappa^{n-1}-1)/(\kappa -1)}-{\left[P_{<}(k)\right]}^{k(\kappa^{n}-1)/(\kappa -1)}\;=\frac{{\left[P_{<}(k)\right]}^{k\kappa^{n-1}/(\kappa -1)}-{\left[P_{<}(k)\right]}^{k\kappa^{n}/(\kappa -1)}}{{\left[P_{<}(k)\right]}^{k/(\kappa -1)}}\equiv \frac{C^{\kappa^{n-1}}-C^{\kappa^{n}}}{C},$$ (B5)

where for simplicity we set $C={\left[P_{<}(k)\right]}^{k/(\kappa -1)}$.

The average distance d(k) can be evaluated as

$$d(k)=\overset{\infty }{\underset{n=1}{\sum }}nP(d=n)=\overset{\infty }{\underset{n=1}{\sum }}n\frac{C^{\kappa^{n-1}}-C^{\kappa^{n}}}{C}\;=\overset{\infty }{\underset{n=0}{\sum }}\frac{C^{\kappa^{n}}}{C}.$$ (B6)

The summation in Eq. (B6) cannot be performed directly. We can approximate its behavior for large k by transforming it into an integral:

$$d(k)≃\frac{1}{C}\int_{0}^{\infty }C^{\kappa^{x}}dx=\frac{1}{C\ln(\kappa )}\int_{1}^{\infty }\frac{C^{z}}{z}dz\;=\frac{\Gamma [0,-\ln(C)]}{C\ln(\kappa )},$$ (B7)

where Γ(a,z) is the incomplete Gamma function [36] and we apply the change of variables $\kappa^{x}=z$. For large k, $C={\left[1-{\left(k/k_{\min}\right)}^{2-\gamma }\right]}^{k/(\kappa -1)}$ tends to 1, so we can expand the incomplete Gamma function in Eq. (B8) for small arguments, Γ(0,z)∼-ln(z) [36], to obtain the asymptotic behavior

$$d(k)∼\frac{-\ln[-\ln(C)]}{C\ln(\kappa )}∼\frac{\ln\left[{\left(\frac{k}{k_{\min}}\right)}^{\gamma -3}\frac{\kappa -1}{k_{\min}}\right]}{\ln(\kappa )},$$ (B8)

where we expand C for large k. We therefore observe the asymptotic behavior for large k in infinite networks as

$$d(k)∼1+\frac{\gamma -3}{\ln(\kappa )}\ln\left(\frac{k}{k_{\min}}\right),$$ (B9)

where the term 1 accounts for the minimum possible distance between nodes.

This calculation, performed in the tree approximation, captures nevertheless the behavior in real uncorrelated power-law networks generated with the UCM [35]. In Fig. 9, we present the result of numerical simulations, together with the numerical evaluation of the summation in Eq. (B6), performed using a discrete power-law degree distribution $P(k)=k^{-\gamma }/[\zeta (\gamma ,k_{\min})-\zeta (\gamma ,k_{\max})]$, where ζ(s,a) is the Hurwitz zeta function [36]. The dashed line represents the result for an infinite network ($k_{\max}=\infty$), while the dot-dashed lines mark the value for networks with maximum degree $k_{\max}=N^{1/(\gamma -1)}$ [37]. The dotted line shows the asymptotic behavior obtained in Eq. (B9).

## APPENDIX C: CUMULATIVE MERGING PERCOLATION ON STRETCHED EXPONENTIAL NETWORKS

Let us consider the example of a network with cumulative degree distribution [27]

$$P_{c}(k)=e^{-k^{\beta }+k_{\min}^{\beta }},$$ (C1)

corresponding to a stretched exponential degree distribution

$$P(k)=-\frac{dP_{k}(k)}{dk}=\beta k^{\beta -1}e^{k_{\min}^{\beta }-k^{\beta }}.$$ (C2)

Applying the extreme value theory, for a finite network of size N we have

$$k_{\max}∼[\ln(N){]}^{1/\beta }.$$ (C3)

The other relevant quantities for CMP are

$$\frac{N_{a}}{N}=\int_{k_{a}}^{\infty }dkP(k)=e^{k_{\min}^{\beta }-k_{a}^{\beta }}$$ (C4)

and

$$P_{a}=\frac{1}{k_{c}}=\int_{k_{a}}^{\infty }dk\frac{kP(k)}{\left\langle k\right\rangle }=\frac{\Gamma \left(1+\frac{1}{\beta },k_{a}^{\beta }\right)}{\Gamma \left(1+\frac{1}{\beta },k_{\min}^{\beta }\right)}$$ (C5)

$$≃\frac{e^{-k_{a}^{\beta }}k_{a}}{\Gamma \left(1+\frac{1}{\beta },k_{\min}^{\beta }\right)},$$ (C6)

where we develop the numerator in the limit of large $k_{a}$. The average number of active neighbors for each active node is

$$\frac{N}{N_{a}}\int_{k_{a}}^{\infty }dkkP(k)P_{a}=\frac{e^{k_{a}^{\beta }}\Gamma {\left(1+\frac{1}{\beta },k_{a}^{\beta }\right)}^{2}}{\Gamma \left(1+\frac{1}{\beta },k_{\min}^{\beta }\right)}\;∼\frac{e^{-k_{a}^{\beta }}k_{a}^{2}}{\Gamma \left(1+\frac{1}{\beta },k_{\min}^{\beta }\right)},$$ (C7)

where we expand the last expression in the limit of large $k_{a}$. Therefore, the average number of active neighbors of an active node vanishes exponentially. This result implies that the size of the DOPGC decays exponentially fast and the extended DOP mechanism is not at work: Small clusters are at a distance much larger than 2 from the DOPGC.

The only mechanism leading to the formation of the CMPGC is the second one, based on the interaction at a distance among isolated nodes. This interaction involves a scale $k_{x}=\omega k_{a}$, such that $r(k_{x})=d(k_{x})$, to ensure that all nodes with $k>k_{x}$ see each other and can merge in the same cluster. To compute d(k), from Appendix B we must evaluate, in the limit of large k,

$$d(k)∼\frac{-\ln\left[-\ln(C)\right]}{C\ln(\kappa )},$$ (C8)

with $C={\left[P_{<}(k)\right]}^{k/(\kappa -1)}$ and κ the branching factor. In this case,

$$P_{<}(k)=1-P_{c}(k)=1-e^{k_{\min}^{\beta }-k^{\beta }}.$$ (C9)

For large k,

$$-\ln(C)≃\frac{k}{\kappa -1}e^{k_{\min}^{\beta }-k^{\beta }},$$ (C10)

and

$$-\ln\left[-\ln(C)\right]≃k^{\beta }-k_{\min}^{\beta }-\ln\left(\frac{k}{\kappa -1}\right).$$ (C11)

Therefore, for large k,

$$d(k)≃\frac{k^{\beta }}{\ln(\kappa )},$$ (C12)

where we disregard constant and logarithmic terms. For $r(k)=k/k_{a}$, from $d(k_{x})=r(k_{x})$, we obtain

$$\omega =\frac{\omega^{\beta }k_{a}^{\beta }}{\ln(\kappa )},$$ (C13)

leading to

$$\omega ={\left(\frac{k_{a}^{\beta }}{\ln(\kappa )}\right)}^{1/(1-\beta )}.$$ (C14)

So, we have

$$k_{x}=k_{a}\omega ={\left(\frac{k_{a}}{\ln(\kappa )}\right)}^{1/(1-\beta )}.$$ (C15)

As a consequence,

$$S_{2}=\int_{k_{x}}^{\infty }dkP(k)=e^{k_{\min}^{\beta }-k_{x}^{\beta }}$$ (C16)

$$\approx e^{k_{\min}^{\beta }-[k_{a}/\ln(\kappa ){]}^{\beta /(1-\beta )}}.$$ (C17)

## APPENDIX D: APPLICATION TO SIS ON STRETCHED EXPONENTIAL NETWORKS

Since the asymptotic behavior of the order parameter for the CMP transition is given by $S_{2}$, the effective finite-size threshold is given by the condition $k_{x}≃k_{\max}$, that is,

$$k_{a}≃\ln(\kappa )k_{\max}^{1-\beta }.$$ (D1)

Using $k_{a}=a(1/\lambda {)}^{2}\ln(1/\lambda )$, this equation implies

$$a(1/\lambda_{c}{)}^{2}\ln(1/\lambda_{c})≃\ln(\kappa )k_{\max}^{1-\beta }.$$ (D2)

Disregarding logarithmic factors, this expression can be inverted, leading, in the limit of large $k_{\max}$, to

$$\lambda_{c}≃\sqrt{\frac{a(1-\beta )}{2\ln(\kappa )}}{\left[k_{\max}^{\beta -1}\ln(k_{\max})\right]}^{1/2}.$$ (D3)

For a stretched exponential degree distribution, $k_{\max}≃\;{\left[\ln(N)\right]}^{1/\beta }$, so we finally have

$$\lambda_{c}≃\sqrt{\frac{a(1-\beta )}{2\beta \ln(\kappa )}}{\left[\ln(N)\right]}^{\left(\beta -1)/(2\beta \right)}{\left\{\ln[\ln(N)]\right\}}^{1/2}.$$ (D4)

In this way, we recover the exact logarithmic dependence of the effective threshold on N for the stretched exponential case, recently found in Ref. [27].

In the limit of a pure exponential distribution, β=1, the previous arguments cannot be applied. However, recent results in Ref. [45] show that the threshold in this case is finite.
